# Intradural non-calcified thoracic disc herniation causing spontaneous intracranial hypotension: a case report

**DOI:** 10.1186/s12893-019-0527-3

**Published:** 2019-06-21

**Authors:** Michael Fiechter, Alexander Ott, Jürgen Beck, Astrid Weyerbrock, Jean-Yves Fournier

**Affiliations:** 1 Department of Neurosurgery, Cantonal Hospital St. Gallen, St. Gallen, Switzerland; 2 Department of Anesthesiology, Cantonal Hospital St. Gallen, St. Gallen, Switzerland; 3 Department of Neurosurgery, Inselspital, University Hospital, University of Bern, Bern, Switzerland

**Keywords:** Cerebrospinal fluid leak, Disc herniation, Non-calcified, Spontaneous intracranial hypotension, Surgical management

## Abstract

**Background:**

Spontaneous intracranial hypotension (SIH) is a rare pathology caused by a cerebrospinal fluid (CSF) leak. If intractable by conventional methods (i.e. bedrest, analgesics, or epidural blood patching) it may lead to the inability of the patient to cope with daily life and eventually to life-threatening complications. Recently, calcified discogenic microspurs or dorsal osteophytes were identified as a major cause for ventral CSF loss through vertical longitudinal dural slits. We report a rare case of intractable SIH due to an intradural disc herniation at the thoracolumbar junction (without signs of calcification) and its management.

**Case presentation:**

A 46-year old woman suffered from orthostatic headache (sudden onset, no history of trauma) due to intractable SIH for over 2 month (without neurologic deficits). There was no clinical amelioration by conservative measures (analgesics, bedrest) and serial unspecific epidural blood patches (repeated for 3 times). She was diagnosed with an intradural disc herniation at the thoracolumbar junction causing a CSF leak. Surgical exploration by a translaminar and transdural approach with removal of the disc herniation and closure of the CSF leak was performed with immediate cessation of orthostatic symptoms. Histological workup revealed non-calcified intervertebral disc material. After 3 months of follow-up and no evidence for clinical relapse the patient returned to work.

**Conclusions:**

We report the rare phenomenon of an intradural non-calcified disc sequester at the thoracolumbar junction as the cause of a ventral dural tear leading to a CSF leak with intractable SIH. This is of particular interest as the major cause of ventral dural leakage is thought to arise from calcified discogenic microspurs or dorsal osteophytes. Furthermore, we comprehensively describe a short and reasonable diagnostic and surgical approach of this rare pathology, which may particularly be of use in daily clinical routine in neurological wards and general surgical spine centers not facing such pathologies on a regular basis.

**Electronic supplementary material:**

The online version of this article (10.1186/s12893-019-0527-3) contains supplementary material, which is available to authorized users.

## Background

Spontaneous intracranial hypotension (SIH) is a rare, usually benign disease caused by a cerebrospinal fluid (CSF) leak [1, 2]. Usually, the exact cause and location of the CSF loss remains idiopathic. Various mechanisms such as minor trauma or nerve root cysts may lead to dural leakage [3]. Recently, calcified discogenic microspurs or dorsal osteophytes were identified as a major cause for ventral CSF loss through vertical longitudinal dural slits (71% of patients with intractable SIH, [4]). Fortunately, most cases of SIH are self-limiting or can successfully be treated with conservative measures such as bedrest and analgesics or minimal invasive procedures such as (serial) epidural blood patching. However, patients with intractable SIH may become incapable to cope with daily life or eventually develop life-threatening pathologies (i.e. subdural hematoma, [5]). In such cases, an extensive workup with advanced imaging methods to identify the origin of CSF leakage and a microsurgical strategy with exploration of potential CSF leakage points is warranted. In our case, a non-calcified intradural disc herniation at the thoracolumbar junction (with no evidence of a microspur/osteophyte) caused CSF leakage.

The aim of this case is to report the rare phenomenon of an intradural disc sequester at the thoracolumbar junction (without signs of calcification) leading to a CSF leak with intractable SIH. Furthermore, we describe the diagnostic workup and stepwise surgical approach of this rare disease and discuss the relevant literature.

## Case presentation

**Fig. 1:**
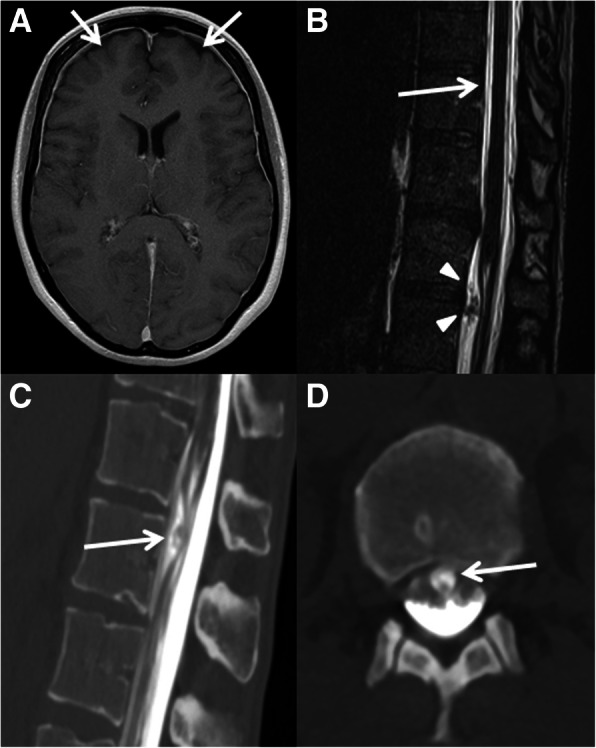
Diagnostic findings in a patient with spontaneous intracranial hypotension (SIH). Initial head-MRI revealed minor bifrontal hygroma (axial view, arrows) with diffuse dural contrast-enhancement as typical findings in a patient with SIH (Panel **a**). Subsequent whole-spine MRI (sagittal view) shows an extradural fluid collection (arrow) and an unidentified trans−/intradural mass lesion (arrowheads) at the level of thoracic vertebrae 12/lumbar vertebrae 1 (Th12/L1, Panel **b**). Finally, CT-myelography confirmed the suspected dural leakage caused by the unidentified trans−/intradural mass lesion (arrow) at the level of Th12/L1 (Panel **c** and **d** with sagittal and axial slices, respectively)

**Fig. 2:**
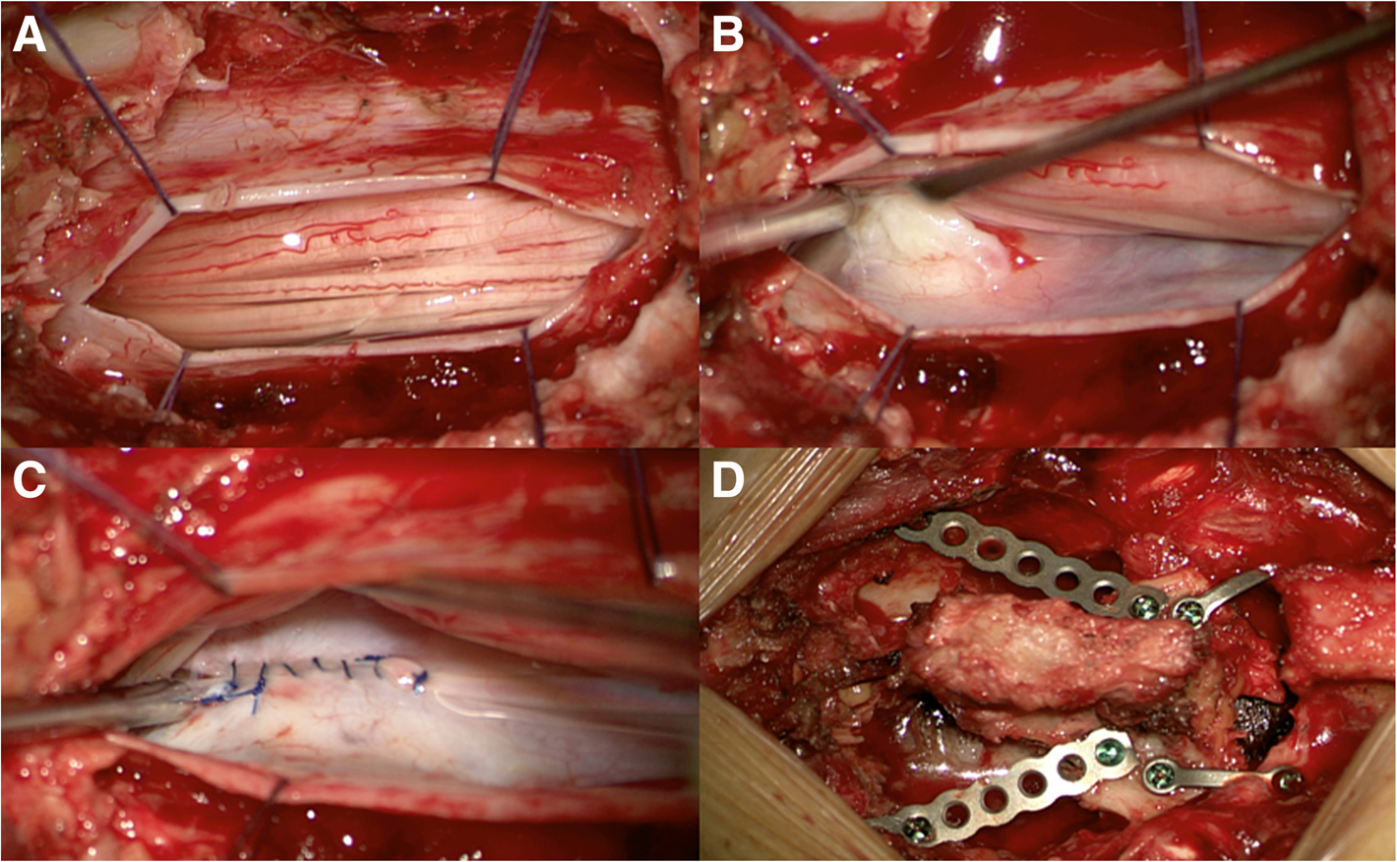
Dorsal transdural approach and removal of an intradural non-calcified disc sequester causing a CSF leak. After laminectomy of thoracic vertebrae 12 (Th12), a dorsal dural opening was performed (Panel **a**). Subsequently, medial mobilisation of the spinal cord (under continuous intraoperative neurophysiological monitoring) revealed an intradural disc sequester at the location of the dural leakage (Panel **b**). After resection of the disc sequester, a water-tight ventral (and dorsal) dural closure was conducted (Panel **c**). Finally, dorsal dural closure and laminoplasty of Th12 concluded the intervention (Panel **d**)


A 46-year old woman initially presented with progressive orthostatic headache (sudden onset) at the emergency unit since 1 month. There was no history of trauma and the neurological exam was normal. Analgesia and bedrest, as prescribed from the patient’s family doctor, had only minor clinical effect. Initial cranial computed tomography (CT) scan revealed small bifrontal hygroma. Pressure measurement by lumbar puncture was considered not reliable due to pressure values equaling to zero. Further workup by cranial and whole-spine magnetic resonance imaging (MRI; fluid-sensitive and thin-sliced) revealed cranial dural contrast-enhancement (with small bifrontal hygroma, Fig. 1a) as well as extradural fluid collection at the level of the thoracic spine suggestive of a dural leak causing CSF loss and thus SIH (Fig. 1b) [5, 6]. Three consecutive (unspecific) lumbar epidural blood patches (serially performed at 3 day intervals) were conducted without sustainable clinical amelioration. Finally, a longspine CT-myelography (dynamic myelography with postmyelography spine CT imaging) identified a possible dural tear due to ventral extradural contrast leakage at the level of the thoracic vertebrae 11/12 and thoracic vertebrae 12 (Th12) / lumbar vertebrae 1 (L1) with high suspicion of a trans−/intradural lesion (Fig. 1c and d). Consequently, surgical exploration of the described levels was performed by a translaminar and transdural approach at the level of Th12 (Fig. 2a-d). Intraoperatively, a ventral dural slit was identified with CSF leakage to the extradural space at the level of Th12/L1 due to an intradural disc sequester (Fig. 2b). Subsequent resection of the disc sequester (by use of sensory/motor evoked potentials, SEPs/MEPs, of lower extremities and anal sphincter) with tight ventral/dorsal dural closure by suture (Fig. 2c) and laminoplasty of Th12 (Fig. 2d) was successfully conducted (refer to the intraoperative video animation in the “Additional file 1”). Histological workup of the resected lesion confirmed non-calcified intervertebral disc material. The postoperative clinical course was uneventful. The patient underwent stepwise and cautious mobilisation to avoid relapse of CSF leakage. Immediate cessation of the orthostatic symptoms was observed postoperatively and at a 3 months follow-up and the patient was able to return to work. Conventional radiography of the thoracolumbar junction showed no signs of dislocation of the artificially reattached lamina of Th12. Written informed consent was obtained from the patient to report and publish individual patient data.

**Additional file 1:** (intraoperative video). Stepwise self-explanatory intraoperative video clip demonstrating the removal of an intradural disc sequester at the thoracolumbar junction causing a dural leakage by a dorsal transdural approach at the level of thoracic vertebrae 12 (Th12). (MP4 25204 kb)


## Discussion and conclusions

We present a rare case of intractable SIH caused by a intradural non-calcified disc herniation at the thoracolumbar junction and its successful surgical treatment. A posterior transdural approach was chosen with subsequent laminoplasty. The posterior transdural approach was considered superior over the anterior approach while offering a wider range of exploration options. For safety reasons, intraoperative neurophysiological monitoring (MEPs/SEPs) was applied during manipulation of the spinal cord.

According to the international headache society, the diagnostic criteria of SIH are considered as follows [7]: A, orthostatic headache; B, the presence of at least one of the following: low CSF opening pressure (≤ 60 mmH_2_O), enduring improvement of clinical symptoms after epidural blood patching, detection of an active spinal CSF leak, typical changes of intracranial hypotension in cranial MRI (e.g., pachymeningeal enhancement or brain sagging); C, absence of a recent history of lumbar (dural) puncture; and D, exclusion of any other potentially attributable disorder. As orthostatic headache and low pressure opening were present in this patient, further diagnostic workup was performed stepwise and began with head- and whole-spine MRI (fluid-sensitive, thin-sliced). After failure of conservative therapeutic measures (bedrest and threefold epidural blood patching), a CT-myelography (dynamic myelography with postmyelography spine CT imaging) was justified. As in other spine pathologies, this is regarded as a secondary diagnostic measure due to its invasiveness and significant radiation exposure of the patient (approx. 10 mSv, [8]). After localisation of the level suspicious for CSF leakage, a surgical exploration was legitimate.

The diagnostic algorithm described by Beck et al. [4] was slightly adapted with omission of measurement of the optic nerve sheath diameter (by ultrasound, [9]) and determination of the CSF outflow resistance (by lumbar infusion testing, [10]). Due to clearly visible extradural fluid accumulation in the whole-spine MRI (at the thoracic/lumbar level) a CSF leak was the only reasonable explanation for the patient’s symptoms. Therefore, no further confirmatory tests were needed. However, in cases with unclear findings in the MRI, these (above mentioned) techniques may provide further confirmation before performing a CT-myelography as one of the most accurate minimal-invasive tests for localisation of a CSF leak.

Fortunately, most patients with SIH can successfully be treated with conservative measures (bedrest, high fluid intake, and pharmaceutical measures such as analgesics or caffeine) or minimal-invasive procedures such as epidural blood patches. This is either performed by unspecific administration of autologous blood at the level L3-L4 (20–40 ml) or, if known, by specific injection of autologous blood at the leakage site. The procedure can optionally be repeated several times at various intervals (as needed). The success rate of epidural blood patches with significant reduction of symptoms within a week and mostly complete remission after 1 month is approximately 83% [11]. However, if unsuccessful (even after several repetitions, [12]) it leaves behind severely impaired patients with intractable SIH. In such cases, diagnostic measures have to be exhausted and if a suspicious site of dural leakage is identified, surgical exploration is required. By the use of intraoperative neurophysiological monitoring during manipulation of the spinal cord we consider a dorso-ventral surgical exploration as a rather safe procedure for the patient.

Interestingly, neither calcified discogenic microspurs or dorsal osteophytes were detected on imaging nor signs of calcification were confirmed by histological workup of the removed intervertebral disc material. This is in slight contrast to the study by Beck et al. [4] in which a calcified microspur or a dorsal osteophyte was identified in all cases of ventral dural leaks. Thus, a simple disc herniation in combination with probably an increased range of motion (as at the thoracolumbar junction) might be sufficient to cause a dural tear (by repetitive microtrauma). A similar case describing a patient with cranial neuropathy due to an intradural disc herniation at the thoracolumbar junction was reported by Rapoport et al. [13]. However, in contrast to our case it appears that there was a traumatic event causing the herniation (“popping event while lifting a heavy object”). Further, whether the disc material was calcified or not has not been investigated.

Despite the fact that this is a single case report (with its obvious limitation as such), we suggest that patients with intractable SIH should undergo surgical exploration of suspicious areas of CSF leakage at the level of the thoracolumbar junction even if discogenic microspurs or dorsal osteophytes have not been identified on imaging.

With this case report we describe the rare phenomenon of an intradural non-calcified disc sequester at the thoracolumbar junction as the cause of a ventral dural tear leading to a CSF leak with intractable SIH. This is of particular interest as the major cause of ventral dural leakage is thought to arise from calcified discogenic microspurs or dorsal osteophytes. Furthermore, we comprehensively describe a short and reasonable diagnostic and surgical approach of this rare pathology, which may particularly be of use in daily clinical routine in neurologic wards and general surgical spine centers not facing such pathologies on a regular basis.

## Data Availability

Further supporting data is available upon request to the corresponding author as long as it does not lead to the identification of the patient or violate any state law with regard to data confidentiality.

## Abbreviations

CSF: Cerebrospinal fluid
CT: Computer tomography
L: Lumbar
MEP: Motor evoked potentials
MRI: Magnetic resonance imaging
SEP: Sensory evoked potentials
SIH: Spontaneous intracranial hypotension
Th: Thoracic
